# Noise-Induced Transitions in a Nonsmooth Producer–Grazer Model with Stoichiometric Constraints

**DOI:** 10.1007/s11538-020-00733-y

**Published:** 2020-04-29

**Authors:** Sanling Yuan, Dongmei Wu, Guijie Lan, Hao Wang

**Affiliations:** 1 College of Science, University of Shanghai for Science and Technology, Shanghai, 200093 China; 2 Department of Mathematical and Statistical Sciences, University of Alberta, Edmonton, AB T6G 2G1 Canada

**Keywords:** Stoichiometric producer–grazer model, Noise-induced state switching, Stochastic sensitivity, Confidence domains, 22E46, 53C35, 57S20

## Abstract

Stoichiometric producer–grazer models are nonsmooth due to the Liebig’s Law of Minimum and can generate new dynamics such as bistability for producer–grazer interactions. Environmental noises can be extremely important and change dynamical behaviors of a stoichiometric producer–grazer model. In this paper, we consider a stochastically forced producer–grazer model and study the phenomena of noise-induced state switching between two stochastic attractors in the bistable zone. Namely, there is a frequent random hopping of phase trajectories between attracting basins of the attractors. In addition, by applying the stochastic sensitivity function technique, we construct the confidence ellipse and confidence band to find the configurational arrangement of equilibria and a limit cycle, respectively.

## Introduction

Ecological stoichiometry is the study of the balance of energy (carbon or C) and multiple nutrients (such as phosphorus or P, nitrogen or N) in ecological interactions (Sterner and Elser 2002). Organisms are composed of key elements, including C, N, P, whose balance affects organismal reproduction and growth, nutrient cycling, and trophic interactions. Plants can be easily limited by nutrient, and herbivores are more nutrient-rich organisms than plants (Elser et al. 2000). The plant nutrient quality can dramatically affect the growth of herbivorous grazers and may even lead to their extinction (Urabe et al. 2002). Historic predator-prey models only consider energy (carbon) flow in the form of population or density. In reality, producer–grazer interaction models should incorporate both food quantity and quality.

In many ecosystems, trophic interactions can be regulated by excessively enriching or limiting some key resources for reproduction and growth (Grover 2002; Peace 2015). To better understand how nutrient availability affects the population reproduction and growth, a series of newly emerged stoichiometric population models have been proposed in the past two decades for studying the underlying laws of ecological stoichiometry (Andersen 1997; Hessen and Bjerkeng 1997; Kuang et al. 2004; Wang et al. 2007, 2008, 2012; Stecha et al. 2012; Jiang et al. 2019; Song et al. 2019; Zhao et al. 2020), and one of the well received stoichiometric producer–grazer models that track both the quantity and the quality of producer is formulated in (Loladze et al. 2000):

1.1 $$\begin{aligned} {\left\{ \begin{array}{ll} \frac{\mathrm {d}x}{\mathrm {d}t}=bx\big (1-\frac{x}{\min \{K,(P-\theta y)/q\}} \big )-f(x)y,\\ \frac{\mathrm {d}y}{\mathrm {d}t}=e\min \left\{ 1,\frac{(P-\theta y)/x}{\theta }\right\} f(x)y-dy, \end{array}\right. } \end{aligned}$$

where *x*, *y* are the densities of producer (phytoplankton/algae) and grazer (daphnia) (mg C/l), respectively; *b* is the intrinsic growth rate of producer (/day); *K* is the carrying capacity of producer, which is positively related to light intensity; *e* is the maximal production efficiency of grazer (no unit); *d* is the specific loss rate of grazer that includes metabolic losses and death (/day); *q* is the minimal phosphorus/carbon ratio in producer (mg P/mg C); $\theta$ is the constant phosphorus/carbon ratio in grazer (mg P/mg C); *P* the total mass of phosphorus in the entire system (mg P/l); *f*(*x*) is the consumption rate of grazer, which is usually one of Holling-type functional responses. By applying the Liebig’s Law of Minimum, the producer’s growth rate is limited by both light and nutrient, and the grazer’s growth rate is limited by both food quantity and food quality. Hence, the growth terms have minimum functions $\min \big (K,\frac{P-\theta y}{q}\big )$ and $e\min \big (1,\frac{(P-\theta y)/x}{\theta }\big )$.

Model (1.1) has complex dynamics such as multiple positive equilibria and bistability (Loladze et al. 2000). Later, Li et al. (2011) provided a rigorous mathematical analysis for global stability results of all equilibria and the existence of limit cycles with Holling-type functional responses and fixed parameters except *K*, and Xie et al. (2018) presented complete global and bifurcation analyses for model (1.1) with Holling type II functional response with all flexible parameters. They found that the model has four types of bistability: between an internal equilibrium and a limit cycle, between an internal equilibrium and a boundary equilibrium, between two internal equilibria, and between a boundary equilibrium and a limit cycle.

In nature, deterministic systems are inevitably affected by various environmental noises which can be important or even dominant in controlling dynamics of trophic interactions. Environmental noises can change the qualitative behavior of a deterministic model (Zhao et al. 2015; Xu and Yuan 2016; Yu et al. 2018, 2019a, b; Zhao and Liu 2019; Wang and Liu 2019; Yu and Yuan 2020). Over the past several decades, many deterministic models with noise-induced transitions have been extensively studied (Xu et al. 2016; Bashkirtseva et al. 2010; Xu et al. 2018; Wu et al. 2019). In Xu et al. (2016), noise perturbations change the coexistence state to extinction for a chemostat model. A new method based on the stochastic sensitivity functions (SSF) technique has been proposed in Bashkirtseva et al. (2010) to construct the analytical description of randomly forced equilibria and cycles of discrete-time models. The presence of coexisting attractors under random perturbations can generate new dynamic regimes, which have no analogues in the deterministic case (Anishchenko et al. 2007; Pisarchik and Feudel 2014). Effects of perturbations in limit cycles were studied in Kurrer and Schulten (1991); Baras (1997). Nonlinear dynamical models show various new phenomena, such as stochastic resonance (Gammaitoni et al. 2009; McDonnell et al. 2010), noise-induced transitions (Horsthemke and Lefever 1984), noise-induced order (Matsumoto and Tsuda 1983; Gassmann 1997), noise-induced chaos (Gao et al. 1999), and noise-induced complexity (Zaks et al. 2005). Analysis of the noise effects on dynamical systems with multiple stable states attracts the attention of many researchers (Kim et al. 1998). Multistable systems exhibit complex dynamics with noise-induced hopping between coexisting attractors and their basins of attraction (Kraut and Feudel 2002; de Souza et al. 2007; Dykman et al. 1990, 1994). The sensitivity analysis of randomly forced oscillations is pivotal for investigating these transitions.

The aim of this paper is to study the phenomena of noise-induced transitions for model (1.1) with Holling-type II functional response by using the SSF technique. The rest of this paper is organized as follows. In Sect. 2, we review the deterministic producer–grazer model with stoichiometric constraints and propose its stochastic version. The analysis of noise-induced transitions and the construction of confidence ellipses for this model will be presented in Sect. 3. In Sect. 4, we provide the construction of both confidence band and confidence ellipse and show that noise-induced transitions occur when confidence domains are intersected. Finally, we conclude and discuss the paper in Sect. 5.

## Model Formulation and Main Results

In this section, in order to formulate our stochastic model, we first recall the main results of model (1.1) with Holling-type II functional response from Xie et al. (2018). As in Xie et al. (2018), we always assume that in this paper

2.1 $$\begin{aligned} e<1, ~q<\theta , ~\frac{ad}{ce-d}<p, ~K\le \min \Bigg \{\frac{P}{q},\frac{\theta }{q}\frac{ad}{ce-d}\Bigg \}, \end{aligned}$$

where $p:=\frac{P}{\theta }$. Then, model (1.1) is simplified as:

2.2 $$\begin{aligned} {\left\{ \begin{array}{ll} \frac{\mathrm {d}x}{\mathrm {d}t}=bx\big (1-\frac{x}{K} \big )-\frac{cxy}{a+x},\\ \frac{\mathrm {d}y}{\mathrm {d}t}=\frac{cey}{a+x}\min \{x,p-y\}-dy. \end{array}\right. } \end{aligned}$$

Denote

$$\begin{aligned} \varOmega =\{(x,y):0<x<K,0<y<p,qx+\theta y<P\}. \end{aligned}$$

Then, $\varOmega$ is a positively invariant set for model (2.2) [see Li et al. (2011) for the detailed proof]. Obviously, $\varOmega$ is an open trapezoid due to the limitation of (2.1). We refer the readers to Fig. 2 in Xie et al. (2018), where the grazer nullcline (red curves) in the forward invariant region $\varOmega$ is the positive *x*-axis and a polygonal line consisted of two line segments: $x=x^*, ~y\in [0, p-x^*]$ (denoted by $l_1$) and $dx+cey=cep-ad,~x\in [x^*,\min \{K,\frac{cep}{d}-a\}]$ (denoted by $l_2$), where $x^*:=\frac{ad}{ce-d}$; the producer nullcline (blue curves) in the forward invariant region $\varOmega$ is the positive *y*-axis and a parabola arc: $y=\frac{b}{c}(1-\frac{x}{K})(a+x)$, $(x,y)\in \varOmega$. Though the vector field defined by system (2.2) is not $C^1$ in $\varOmega$, it is locally Lipschitz-continuous, which guarantees the existence and uniqueness of solutions of system (2.2).

System (2.2) always has two boundary equilibria: $E_{0}=(0,0)$ and $E_{1}=(K,0)$. It may also have none or one to three coexistence/interior equilibria. The dynamics of system (2.2) is completely determined by some critical values about *K* listed below [see Xie et al. (2018) for more details]:

- $$K_1=x^{*}=\frac{ad}{ce-d}$$;
- $$K_2=a+2x^{*}$$;
- $$K_3=\frac{x^{*}}{1-\frac{c}{b}\cdot \frac{p-x^{*}}{a+x^{*}}}$$;
- $K_4$ is the value of *K* at which the line segment $l_{2}$ is tangent to the parabola (not always exists);
- $$K_5=\frac{cep}{d}-a$$.

The authors in Xie et al. (2018) have provided a complete global analysis for system (2.2) without fixing any parameter. Their analysis shows that the model has far richer dynamics than those found in the previous paper (Li et al. 2011). For example, system (2.2) may have three interior equilibria $E_2$, $E_3$, $E_4$ in the forward invariant region $\varOmega$ and four types of bistability may appear: (i) between $E_2$ and $E_4$, (ii) between the limit cycle and $E_4$, (iii) between $E_2$ and $E_1$, (iv) between the limit cycle and $E_1$. The readers are referred to Xie et al. (2018) for more details. In this paper, we are only concerned with the first two types and explore the impact of noises existed in the environment on bistability between two interior attractors: one is for the stable equilibrium $E_2$ or the unique stable limit cycle surrounding the unstable equilibrium $E_2$, the other is for the stable internal equilibrium $E_4$.

We remark that a necessary condition for system (2.2) to have three internal equilibria is that $K_4$ exists. Here, we just mention the following two results from Xie et al. (2018) with small modifications, which will be used in the sequel analysis.

### Lemma 2.1

**Fig. 1:**
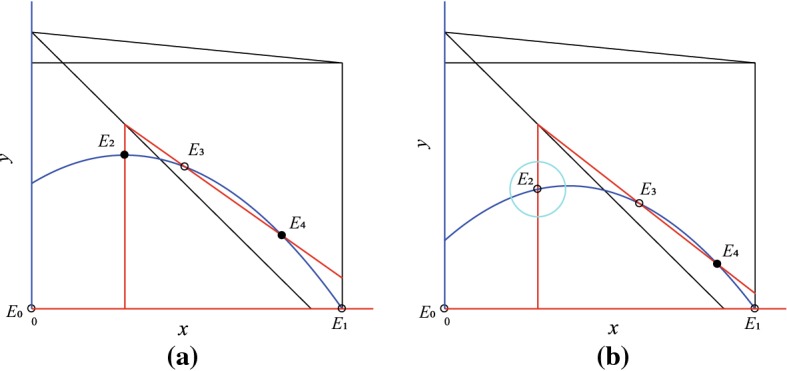
Bistability of model (2.2) occur: **a** two internal equilibria; **b** an equilibrium and a limit cycle. Here, hollow dots denote unstable equilibria, solid dots are stable equilibria; blue closed circle denotes stable limit cycle (Color figure online)

(Theorem 3.2 (3) of Xie et al. (2018)) Assume that $K_1< K_4< K_2< K_3 < K_5$. If $K\in (K_{4},K_{2})$, there exist three internal equilibria: $E_{2}$ and $E_{4}$ are two stable equilibria, $E_{3}$ is a saddle, and the model has no limit cycle, see Fig. 1a. Hence, bistability occurs: orbits on the left of the stable manifold of $E_{3}$ will eventually tend to $E_{2}$, orbits on the right of the stable manifold of $E_{3}$ will eventually tend to $E_{4}$, and the separatrix is the stable manifold of $E_{3}$. Moreover, the model has three heteroclinic orbits from $E_{3}$ to $E_{2}$, from $E_{3}$ to $E_{4}$, and from $E_{1}$ to $E_{4}$, respectively.

### Lemma 2.2

(Theorem 3.2 (4) of Xie et al. (2018)) Assume that $K_1< K_4< K_2< K_3 < K_5$. If $K\in (K_{2},K_{3})$, there also exist three internal equilibria: $E_{3}$ is a saddle, $E_{4}$ is stable, but $E_{2}$ becomes unstable. Meanwhile, the model has a unique stable limit cycle surrounding the equilibrium $E_{2}$ (cyan dashed-dotted line) for $K\in (K_2, K_*]$, where $K_*$ is the broken value of *K* for the limit cycle, see Fig. 1b. In this case bistability occurs: solutions of the model on the right of the stable manifold of $E_{3}$ tend to the equilibrium $E_{4}$, solutions of the model on the left of the stable manifold of $E_{3}$ tend to the limit cycle, and the separatrix is the stable manifold of $E_{3}$. Moreover, the model has two heteroclinic orbits from $E_{3}$ to $E_{4}$ and from $E_{1}$ to $E_{4}$, respectively.

We are now in a position to propose our stochastic model. From the biological point of view, the sources of environmental noise affecting the producer and the grazer are different. Following the approach used in Refs. Imhof and Walcher (2005) we consider randomness into deterministic system (2.2) and then obtain the following stochastic differential equation:

2.3 $$\begin{aligned} {\left\{ \begin{array}{ll} \mathrm {d}x=\big (bx\big (1-\frac{x}{K} \big )-\frac{cxy}{a+x} \big )\mathrm {d}t+\varepsilon _x x\mathrm {d}B_{1},\\ \mathrm {d}y=\big (\frac{cey}{a+x}\min \{x,p-y\}-dy \big )\mathrm {d}t+\varepsilon _y y\mathrm {d}B_{2}, \end{array}\right. } \end{aligned}$$

where $B_{1}(t)$ and $B_{2}(t)$ are two standard one-dimensional independent Brownian motions, $\varepsilon _x, \varepsilon _y$ are the noise intensities.

For the simplicity of discussion, we assume $\varepsilon _x= \varepsilon _y=\varepsilon$. Notice that system (2.3) consists of two subsystems. Therefore, we assume that $\varepsilon =\varepsilon _{1}$ when $x+y\le p$ and $\varepsilon =\varepsilon _{2}$ when $x+y>p$. The following theorem is about the existence and uniqueness of the global positive solution of model (2.3), whose proof is provided in “Appendix 1”.

### Theorem 2.1

Assume that $\varepsilon _x= \varepsilon _y$. Then, for any given positive initial value $(x(0), y(0))\in {\mathbb {R}}^2_+$, stochastic system (2.3) admits a unique positive solution (*x*(*t*), *y*(*t*)) for $t\ge 0$ and the solution will remain in ${\mathbb {R}}^2_+$ with probability one, in other words, $(x(t),y(t))\in {\mathbb {R}}^2_+$ for all $t\ge 0$ almost surely (a.s.).

In the following, we study the phenomenon of noise-induced transitions between stochastic attractors for stochastic model (2.3).

## Analysis of Noise-Induced Transitions Between Two Internal Equilibria

In order to analyze the influence of noise on model (2.2), we take the following realistic parameter values from (Li et al. 2011):

3.1 $$\begin{aligned} \begin{aligned} e=0.8, b=1.2, d=0.25, \theta =0.04, q=0.004, c=0.8, a=0.25. \end{aligned} \end{aligned}$$

We further take $p=0.615$, it is easy to compute from Example 2 in Xie et al. (2018) that

$$\begin{aligned} K_1=0.1625, K_2=0.5705, K_3=0.6139, K_4=0.5661, K_5=1.3244. \end{aligned}$$

**Fig. 2:**
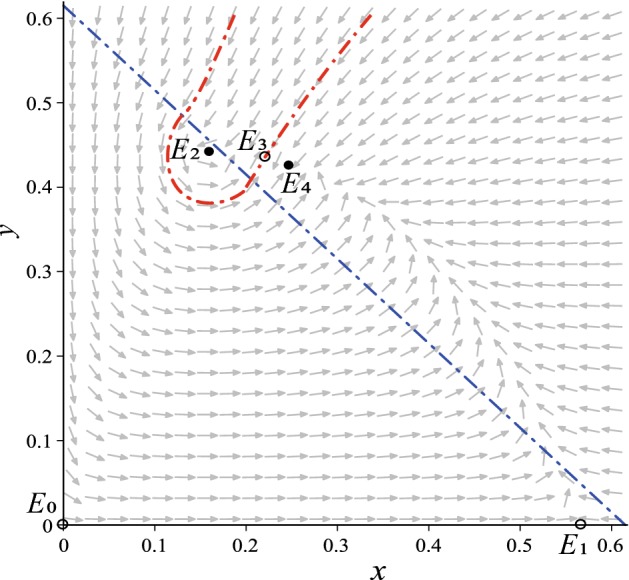
Vector field of the deterministic model and the equation of the separatrix of two subsystems: $x+y=p$ (Color figure online)

Obviously, $K_1<K_4<K_2<K_3<K_5$. Now taking the above parameter values and $K=0.567$ in model (2.2) (*i.e.*, stochastic model (2.3) with $\varepsilon =0$), there exist a washout equilibrium $E_{0}=(0,0)$, a mono-culture equilibrium $E_{1}=(0.5670,0)$, and three coexistence/interior equilibria $E_{2}=(0.1603,0.4415)$, $E_{3}=(0.2193,0.4317)$, and $E_{4}=(0.2454,0.4215)$. It is easy to see that $K\in (K_4, K_2)=(0.5661,0.5705)$. From Example 2 in Xie et al. (2018) and Lemma 2.1, we know that equilibria $E_{0}$ and $E_{1}$ are unstable, $E_{3}$ is a saddle point, coexistence equilibria $E_{2}$ and $E_{4}$ are locally asymptotically stable. Using the command “DEtools [phaseportrait]” of Maple, the vector field of the deterministic model with given parameters is drawn as in Fig. 2, in which the red dash-dotted line is the separatrix of two attraction domains and the blue dash-dotted line is the separatrix of two subsystems.

**Fig. 3:**
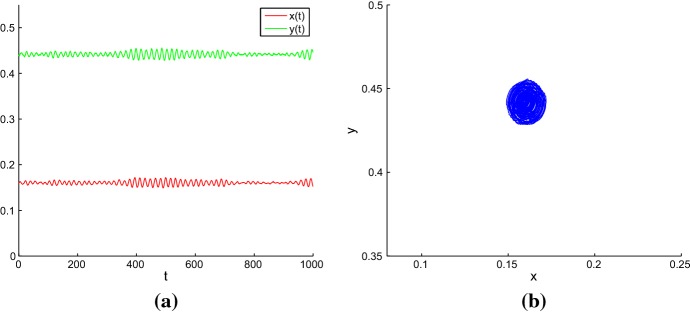
**a** Time series of *x*(*t*) and *y*(*t*); **b** Phase trajectory for stochastic model (2.3) with the initial value (0.1603, 0.4415) and the noise intensity $\varepsilon _{1}=0.0015$ (Color figure online)

**Fig. 4:**
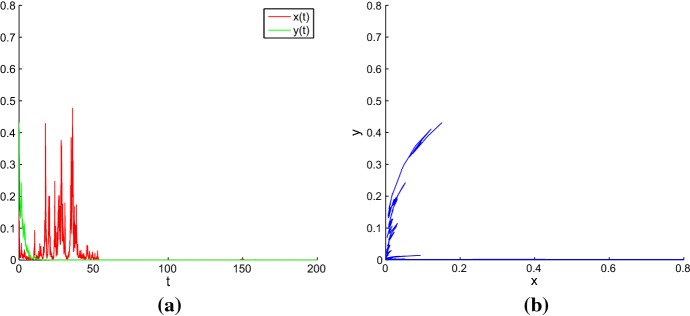
**a** Time series of *x*(*t*) and *y*(*t*); **b** Phase trajectory for stochastic model (2.3) with the initial value (0.1603, 0.4415) and the noise intensity $\varepsilon _{1}=0.15$ (Color figure online)

For the deterministic model, the trajectory with its initial point inside the separatrix converges to the coexistence equilibrium $E_{2}$, and the trajectory with its initial value outside the separatrix converges to the coexistence equilibrium $E_{4}$. However, the dynamics of the stochastic model sometimes can be difficult to predict accurately. In order to illustrate the impact of environmental noise on dynamical behaviors of model (2.2), we take equilibrium $E_{2}$ as an example. For equilibrium $E_{4}$, we can discuss similarly. For a weak noise, the stochastic trajectory with its initial value near the deterministic coexistence equilibrium will fluctuate around this equilibrium, the densities of grazer and producer stay close to their equilibrium values (see Fig. 3). However, as the noise intensity increases and becomes sufficiently large, both grazer and producer can go extinct (see Fig. 4). Hence, there is a critical noise intensity $\varepsilon _1^{*}$, when $0<\varepsilon _1<\varepsilon _1^{*}$, both grazer and producer persist. Furthermore, in this interval we find interesting phenomena of noise-induced transitions between two stochastic attractors. Here, Figs. 3 and 4 are drawn with Matlab software by using the Euler-Maruyama method developed in Ref. Higham (2001). The same method is also used in the numerical simulations of time series and random trajectories in the later figures of the paper.

In the following, by using the SSF method (see “Appendix 2”), we construct confidence ellipses for stochastic model (2.3) to characterize the configurational arrangement of stochastic coexistence equilibria and then further estimate the threshold value of the noise intensity of state switching.

Define

$$\begin{aligned} F=\left( \begin{array}{cccc} \displaystyle f_{11} &{} \displaystyle f_{12}\\ f_{21} &{} \displaystyle f_{22} \\ \end{array}\right) ,~ G=\left( \begin{array}{cccc} \displaystyle g_{11} &{} 0 \\ 0 &{} \displaystyle g_{22} \\ \end{array}\right) ,~ S=GG^{T}, \end{aligned}$$

where

$$\begin{aligned} f_{11}=\frac{cxy}{(a+x)^{2}}-\frac{bx}{K},~ f_{12}=-\frac{cx}{a+x},~ f_{21}=\frac{acey}{(a+x)^{2}},~ f_{22}=0 \end{aligned}$$

and

$$\begin{aligned} g_{11}=x,~ g_{22}=y. \end{aligned}$$

The stochastic sensitivity matrix

$$\begin{aligned} W=\left( \begin{array}{cccc} \displaystyle w_{11} &{} \displaystyle w_{12}\\ w_{21} &{} \displaystyle w_{22} \\ \end{array}\right) \end{aligned}$$

satisfies the following equations:

$$\begin{aligned} \left\{ \begin{array}{l} \displaystyle {2f_{11}w_{11}+f_{12}w_{12}+f_{12}w_{21}=-g_{11}^{2}}, \\ \displaystyle {f_{21}w_{11}+(f_{11}+f_{22})w_{12}+f_{12}w_{22}=0,}\\ \displaystyle {f_{21}w_{11}+(f_{11}+f_{22})w_{21}+f_{12}w_{22}=0,}\\ \displaystyle {f_{21}w_{12}+f_{21}w_{21}+2f_{22}w_{22}=-g_{22}^{2}}. \end{array} \right. \end{aligned}$$

It then follows from (B.3) that the confidence ellipse equation is

3.2 $$\begin{aligned} \begin{aligned} \big \langle (x-{\bar{x}},y-{\bar{y}})^{T},W^{-1}((x-{\bar{x}},y-{\bar{y}})^{T})\big \rangle =2 \varepsilon ^{2}\ln \frac{1}{1-P}, \end{aligned} \end{aligned}$$

where $({\bar{x}}, {\bar{y}})$ is a interior equilibrium of deterministic model (2.2), $\varepsilon$ and *P* are respectively the noise intensity and a fiducial probability.

Taking the same parameters as in Fig. 2, for the interior equilibrium $E_2=(0.1603,0.4415)$ we have

$$\begin{aligned} W=\left( \begin{array}{cccc} \displaystyle 83.1776 &{} \displaystyle -0.5261\\ \displaystyle -0.5261 &{} \displaystyle 111.6737 \\ \end{array}\right) ,~ W^{-1}=\left( \begin{array}{cccc} \displaystyle 0.0120 &{} 0.0001 \\ 0.0001 &{} \displaystyle 0.0090 \\ \end{array}\right) , \end{aligned}$$

respectively. Then, from Eq. (3.2) the confidence ellipse equation of $E_{2}$ is

$$\begin{aligned}&0.0120(x-0.1603)^{2}+0.0002(x-0.1603)(y-0.4415) +0.009(y-0.4415)^{2}\\&\quad =2\varepsilon _{1}^{2}\ln \frac{1}{1-P_{1}}. \end{aligned}$$

**Fig. 5:**
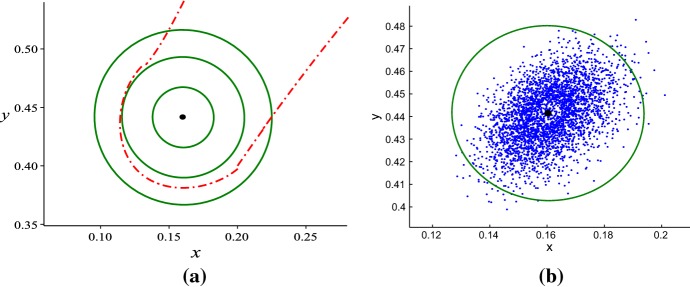
**a** Separatrix (dashed-dotted) and confidence ellipses (solid) for $\varepsilon _{1}=0.001$ (small), $\varepsilon _{1}=0.002$ (middle), $\varepsilon _{1}=0.003$ (large). **b** Random state (blue) of stochastic model (2.3) and confidence ellipse (green) for $\varepsilon _{1}=0.0015$

For fixing fiducial probability $P_{1}=0.95$, we take the noise intensities $\varepsilon _{1}=0.001, 0.002$, and 0.003, respectively, resulting in the corresponding confidence ellipses shown in Fig. 5a. Obviously, as the noise intensity increases, the confidence ellipse starts to expand and after crossing the separatrix, it enters the attraction domain of the coexistence equilibrium $E_{4}$. The value $\varepsilon _{1}$ corresponding to the tangency of the confidence ellipse can be used as an estimation for the threshold noise intensity of the onset of noise-induced transitions. Here, $\varepsilon _{1}=0.002$. Figure 5b illustrates the confidence ellipse with $\varepsilon _{1}=0.0015$, one can see that the random states of the stochastic model are distributed around the corresponding deterministic coexistence equilibrium, and they belong to the interior of the confidence ellipse with probability 0.95.

Similarly, the stochastic sensitivity matrix corresponding to equilibrium $E_{4}$ is

$$\begin{aligned} W=\left( \begin{array}{cccc} \displaystyle 6.5749 &{} \displaystyle -2.7132\\ \displaystyle -2.7132 &{} \displaystyle 1.4468 \\ \end{array}\right) , W^{-1}=\left( \begin{array}{cccc} \displaystyle 0.6726 &{} 1.2613 \\ 1.2613 &{} \displaystyle 3.0565 \\ \end{array}\right) , \end{aligned}$$

then the confidence ellipse equation of $E_{4}$ is

$$\begin{aligned}&0.6726(x-0.2454)^{2}+2.5226(x-0.2454)(y-0.4215)+3.0565(y-0.4215)^{2}\\&\quad =2\varepsilon _{2}^{2}\ln \frac{1}{1-P_{2}}. \end{aligned}$$

**Fig. 6:**
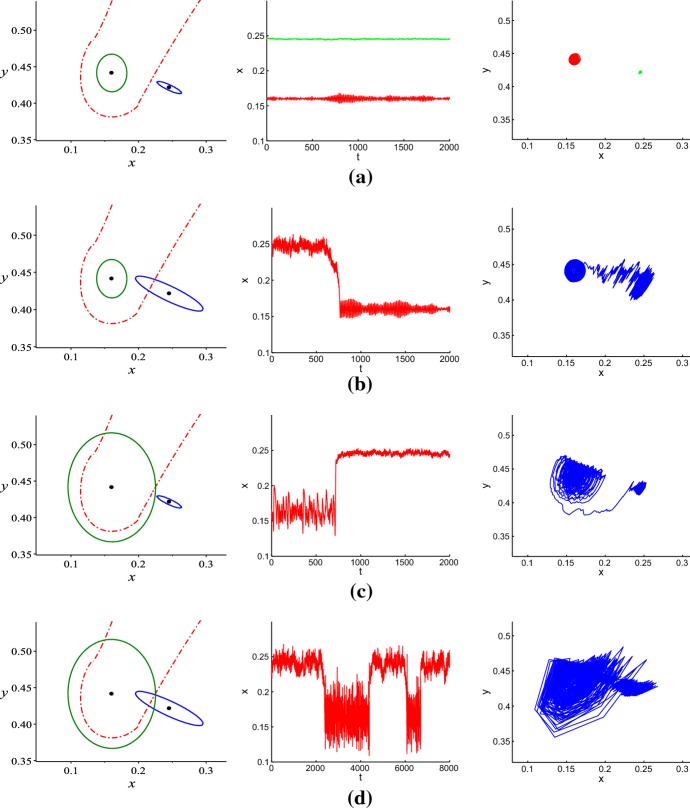
Confidence ellipses (left), time series (middle) and random trajectories (right) of stochastic model (2.3) for: **a**$\varepsilon _{1}=0.001$, $\varepsilon _{2}=0.003$; **b**$\varepsilon _{1}=0.001$, $\varepsilon _{2}=0.008$; **c**$\varepsilon _{1}=0.003$, $\varepsilon _{2}=0.003$; **d**$\varepsilon _{1}=0.003$, $\varepsilon _{2}=0.008$ (Color figure online)

By taking $P_{1}=P_{2}=0.95$, we consider two confidence ellipses together. For a weak noise ($\varepsilon _{1}=0.001, \varepsilon _{2}=0.003$), two confidence ellipses of the coexistence $E_{2}$ (green) and $E_{4}$ (blue) are distinctly separated by the separatrix of two attraction basins (see the left panel of Fig. 6a), and solutions starting from one side will eventually approach the coexistence equilibrium $E_{2}$ (red) or $E_{4}$ (green) on that side (see the middle panel of Fig. 6a). Stochastic trajectories leaving the unforced deterministic attractors concentrate in their small neighborhoods (see the right panel of Fig. 6a). Here, the dynamics of the stochastic model is almost regular and small noises have little impact on the densities of grazer and producer.

As the noise intensity increases, the confidence ellipse expands. We keep $\varepsilon _{1}=0.001$ and increase $\varepsilon _{2}$ to 0.008, then the confidence ellipse (blue) of the coexistence equilibrium $E_{4}$ crosses the separatrix (see the left panel of Fig. 6b). Solutions starting from the attraction basin of $E_{4}$ will eventually approach the coexistence equilibrium $E_{2}$ on the other side with high probability (see the middle panel of Fig. 6b). The phenomenon of noise-induced transition occurs (see the right panel of Fig. 6b). We also have a symmetric result in Fig. 6c (for $\varepsilon _{1}=0.003, \varepsilon _{2}=0.003$).

The above three situations are all related to the initial point. However, when both confidence ellipses expand and cross the separatrix and intersect each other (see the left panel of Fig. 6d for $\varepsilon _{1}=0.003, \varepsilon _{2}=0.008$), we obtain frequent random hopping of phase trajectories between attraction basins of the equilibria $E_{2}$ and $E_{4}$. On the phase plane, a place of this intersection marks a location of the “transition bridge” between basins of attraction where noise-induced transitions are most likely to occur. These results obtained by the confidence domain method are in agreement with the direct numerical simulation of time series (middle panel) and random trajectories (right panel) in Fig. 6d. The difference is that the initial value can be evaluated anywhere in the invariant set.

## Analysis of Noise-Induced State Switching Between Confidence Ellipse and Confidence Band

When $p=0.617$, $K=0.574$ and other parameter values are the same as in (3.1), Lemma 2.2 implies that the coexistence equilibrium $E_{4}=(0.2652,0.4157)$ of model (2.2) is stable, but $E_{2}=(0.2083,0.4380)$ becomes unstable. Meanwhile, the model has a unique stable limit cycle surrounding the equilibrium $E_{2}$, where both population levels fluctuate around a coexistence equilibrium. We next analyze noise-induced state switching by constructing confidence band and confidence ellipse.

In the stochastic case, the limit cycle generally disappears, but the trajectories will remain in a small neighborhood of the deterministic limit cycle for a small noise. For characterizing the configurational arrangement of this neighborhood, in what follows, we will construct the confidence band for stochastic model (2.3).

For convenience, let

$$\begin{aligned} \begin{aligned} F_{1}(x,y)&=bx\bigg (1-\frac{x}{K} \bigg )-\frac{cxy}{a+x},\\ F_{2}(x,y)&=\frac{cexy}{a+x}-dy, \end{aligned} \end{aligned}$$

and denote the deterministic limit cycle by $\varGamma (x(t),y(t)),t\in [0,T]$, where *T* is the period. Then, we can write matrices *F*(*t*), *G*(*t*) and *S*(*t*) as follows:

$$\begin{aligned} F(t)=\left( \begin{array}{cccc} \displaystyle f_{11}(t) &{} \displaystyle f_{12}(t)\\ f_{21}(t) &{} \displaystyle f_{22}(t) \\ \end{array}\right) ,~ G(t)=\left( \begin{array}{cccc} \displaystyle g_{11}(t) &{} 0 \\ 0 &{} \displaystyle g_{22}(t) \\ \end{array}\right) , S(t)=G(t)G(t)^{T}, \end{aligned}$$

where

$$\begin{aligned} f_{11}(t)= & {} \bigg (b-\frac{2bx}{K}-\frac{acy}{(a+x)^{2}}\bigg )\mid _{\varGamma },~ f_{12}(t)=-\bigg (\frac{cx}{a+x}\bigg )\mid _{\varGamma }, \\ f_{21}(t)= & {} \bigg (\frac{acey}{(a+x)^{2}}\bigg )\mid _{\varGamma },~ f_{22}(t)=\bigg (\frac{cex}{a+x}-d\bigg )\mid _{\varGamma }, \end{aligned}$$

and

$$\begin{aligned} g_{11}(t)=x\mid _{\varGamma },~ g_{22}(t)=y\mid _{\varGamma }. \end{aligned}$$

From (B.4), we know that the stochastic sensitivity function $\mu (t)$ satisfies the following boundary problem:

$$\begin{aligned} {\dot{\mu }}=a(t)\mu +b(t),~\mu (0)=\mu (T), \end{aligned}$$

where

$$\begin{aligned} a(t)= & {} 2f_{11}(t)p_{1}^{2}(t)+2(f_{12}(t) +f_{21}(t))p_{1}(t)p_{2}(t)+2f_{22}(t)p_{2}^{2}(t), \\ b(t)= & {} g_{11}(t)p_{1}^{2}(t)+g_{22}(t)p_{2}^{2}(t). \end{aligned}$$

Here,

$$\begin{aligned} p_{1}(t)= & {} \frac{F_{2}(x,y)}{\sqrt{F_{1}^{2}(x,y)+F_{2}^{2}(x,y)}},\\ p_{2}(t)= & {} -\frac{F_{1}(x,y)}{\sqrt{F_{1}^{2}(x,y)+F_{2}^{2}(x,y)}} \end{aligned}$$

are elements of a vector function $p(t)=(p_{1}(t),p_{2}(t))^{T}$ orthogonal to vector $(F_{1}(x,y),F_{2}(x,y))^{T}\mid _{\varGamma }$. It follows from (B.5) that the boundaries $\varGamma _{1,2}(t)$ of the confidence band have the following explicit parametrical form:

$$\begin{aligned} \varGamma _{1}(t)= & {} \varGamma (t)+\varepsilon _{1}k\sqrt{2\mu (t)}p(t),\\ \varGamma _{2}(t)= & {} \varGamma (t)-\varepsilon _{1}k\sqrt{2\mu (t)}p(t). \end{aligned}$$

Here, the parameter *k* is connected with the fiducial probability $P_{1}$ by the formula $k=erf^{-1}(P_{1})$, where $erf(x)=\frac{2}{\sqrt{\pi }}\int _{0}^{x}e^{-t^{2}}dt$ is the error function.

**Fig. 7:**
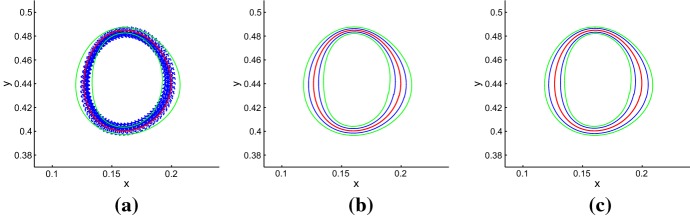
Confidence bands for stochastic model (2.3). **a** Random states (blue) around $\varGamma$ (red) and band (green) for $\varepsilon _{1}=0.0005$, $P_{1}=0.95$; **b**$P_{1}=0.95$ and $\varepsilon _{1}=0.0006$ (green), $\varepsilon _{1}=0.0003$ (blue); **c**$\varepsilon _{1}=0.0006$ and $P_{1}=0.95$ (green), $P_{1}=0.75$ (blue)

In Fig. 7a, the red line is the deterministic limit cycle, the blue points are the random states on different time and the two green lines are the boundaries of the confidence band. Obviously, the random states are distributed around the deterministic limit cycle, and they belong to the interior of the confidence band with probability 0.95.

Figure 7b, c illustrate the effects of the noise intensity and fiducial probability on the size of confidence band. It is easy to see from Fig. 7b, c that the configurational arrangement of confidence band begins to expand as the noise intensity or fiducial probability increases. This result can be deduced from the expressions of $\varGamma _{1}(t)$ and $\varGamma _{2}(t)$.

Following the same logic as in Sect. 3, we can obtain the stochastic sensitivity matrix of $E_{4}$ as follows:

$$\begin{aligned} W=\left( \begin{array}{cccc} \displaystyle 3.2019 &{} -1.4053\\ -1.4053 &{} \displaystyle 0.9516 \\ \end{array}\right) ,\ \text {and}\ W^{-1}=\left( \begin{array}{cccc} \displaystyle 0.8876 &{} 1.3108 \\ 1.3108 &{} \displaystyle 2.9867 \\ \end{array}\right) . \end{aligned}$$

Then, from (B.3) the confidence ellipse equation of $E_{4}$ is

$$\begin{aligned}&0.8876(x-0.2652)^{2}+2.6216(x-0.2652)(y-0.4157)+2.9867(y-0.4157)^{2}\\&\quad =2\varepsilon _{2}^{2}\ln \frac{1}{1-P_{2}}. \end{aligned}$$

**Fig. 8:**
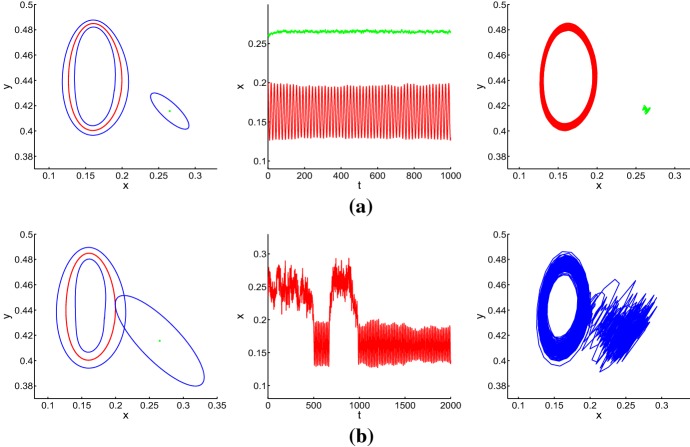
Confidence domains (left), time series (middle) and random trajectories (right) of stochastic model (2.3) for: **a**$\varepsilon _{1}=0.0006$, $\varepsilon _{2}=0.0006$; **b**$\varepsilon _{1}=0.001$, $\varepsilon _{2}=0.015$ (Color figure online)

By taking $P_{1}=P_{2}=0.95$, we investigate the evolution process between confidence band and confidence ellipse. For a weak noise, confidence band and confidence ellipse are distinctly separated, the trajectory with its initial point in either of two attraction basins will eventually approach confidence band or confidence ellipse in that attraction basin. Stochastic trajectories leaving the unforced deterministic attractors concentrate in their small neighborhoods (see Fig. 8a for $\varepsilon _{1}=0.0006, \varepsilon _{2}=0.006$). With $\varepsilon _{1}=0.001, \varepsilon _{2}=0.015$, the left panel of Fig. 8b illustrates that confidence band and confidence ellipse expand and intersect each other. This leads to frequent random hopping of phase trajectories between attraction basins of the limit cycle and a coexistence equilibrium (see the middle and right panels of Fig. 8b).

## Discussion

We study noise-induced transitions from one coexistence to another coexistence in a constant interval for a stochastically forced producer–grazer model with stoichiometric constraints. The corresponding deterministic model is nonsmooth and its global stability and bifurcation are fully analyzed in Xie et al. (2018). When appropriate parameters are chosen, bistability can occur. The attractors (stable equilibria $E_{2}$, $E_{4}$, or the limit cycle $\varGamma$) correspond to the stable coexistence of grazer and producer. Two attraction basins are distinctly separated by the separatrix, which is the stable manifold of saddle point $E_{3}$, and solutions from either side of separatrix will eventually approach the equilibrium or the limit cycle on that side. However, when there is noise disturbance, the boundary may be damaged by noise, and the solutions in the attractive basin of one stable coexistence equilibrium can eventually approach the other stable coexistence equilibrium with a high probability.

Based on the technique of SSF, we construct confidence ellipses to characterize the phenomenon of noise-induced state switching between two stochastic coexistence equilibria. It provides us the general location of the equilibria and the distribution of random states in the stochastic model. For a weak noise, the confidence ellipses are completely contained in the attraction basins of the corresponding coexistence equilibria, and the random trajectories will not leave the confidence ellipses with a high probability. As the noise intensities increase, the confidence ellipses begin to expand and when only one ellipse crosses the separatrix, we study the phenomenon of noise-induced transitions from a coexistence to another coexistence. Furthermore, when both of confidence ellipses cross the separatrix of two attraction basins and intersect each other, we obtain that frequent random hopping of phase trajectories between the attraction basins of the equilibria.

In addition, we have established the confidence band for the limit cycle of model (2.3), which provides us the general location of the stochastic cycle and the distribution of random states around the deterministic limit cycle. Following the same logic, we analyze the phenomenon of noise-induced transitions via confidence band and confidence ellipse.

Theoretically, the solution of the stochastic model with multi-stability runs long enough to cause state switching. When one confidence domain intersects another, the probability of switching is very high (it can be done in a very short time).

In fact, the phenomenon of noise-induced transitions we get is independent of the way the noise is introduced, we’re just taking one of these cases. For instance, we can introduce randomness into deterministic model (2.2) by perturbing the parameter $c \rightarrow c+\varepsilon \dot{B}(t)$ and obtain the following stochastic differential equation:

$$\begin{aligned} {\left\{ \begin{array}{ll} \mathrm {d}x=\big (bx\big (1-\frac{x}{K} \big )-\frac{cxy}{a+x} \big )\mathrm {d}t-\varepsilon \frac{cxy}{a+x}\mathrm {d}B,\\ \mathrm {d}y=\big (\frac{cey}{a+x}\min \{x,p-y\}-dy \big )\mathrm {d}t+\varepsilon \frac{cey}{a+x}\min \{x,p-y\}\mathrm {d}B. \end{array}\right. } \end{aligned}$$

The results of numerical simulation of time series and random trajectories are similar to those presented in this paper.

We discuss the generality of model (2.2), where noised-induced state switching might not be as common in a real-world situation. There are different noise intensities and ranges for different species. Our work expounded how the confidence domain method is used to understand the qualitative changes in stochastic dynamics at which noise-induced state switching occurs with high probabilities. This method is applicable to nonsmooth competition models and more complex higher dimensional models in aquatic or terrestrial ecosystems. Our results enrich the study of asymptotic behaviors in stoichiometric producer–grazer models and help better understand the stoichiometric producer–grazer dynamics in the stochastic perspective.

## A Proof of Theorem 2.1

### Proof

Without lose of generality, we assume that $x(0)+y(0)>p$ (same logic follows when $x(0)+y(0)\le p$). Let us introduce the stopping time sequence $\{\tau _i\}, i=1,2,\cdots$ as follows:

$$\begin{aligned} \tau _1=\inf \{t\ge 0, x(t)+y(t)\le p\},~~ \tau _2=\inf \{t\ge \tau _1, x(t)+y(t)>p\} \end{aligned}$$

and

$$\begin{aligned} \tau _{2k+1}=\inf \{t\ge \tau _{2k}, x(t)+y(t)\le p\},~~ \tau _{2k+2}=\inf \{t\ge \tau _{2k+1}, x(t)+y(t)>p\} \end{aligned}$$

for $k=1,2,\cdots$. Thus when $t\in [\tau _{2k},\tau _{2k+1})$, model (2.3) becomes

A.1 $$\begin{aligned} {\left\{ \begin{array}{ll} \mathrm {d}x=\left( bx(1-\frac{x}{K})-\frac{cxy}{a+x}\right) dt+\varepsilon _2 xdB_1,\\ \mathrm {d}y=\left( \frac{cey(p-y)}{a+x}-dy\right) dt+\varepsilon _2 ydB_2,\\ \end{array}\right. } \end{aligned}$$

and when $t\in [\tau _{2k+1},\tau _{2k+2})$, model (2.3) becomes

A.2 $$\begin{aligned} {\left\{ \begin{array}{ll} \mathrm {d}x=\left( bx(1-\frac{x}{K})-\frac{cxy}{a+x}\right) dt+\varepsilon _1 xdB_1,\\ \mathrm {d}y=\left( \frac{cexy}{a+x}-dy\right) dt+\varepsilon _1 ydB_2,\\ \end{array}\right. } \end{aligned}$$

where $k=0,1,2,\cdots$ and $\tau _0=0$. Notice that (A.1) and (A.2) both have a unique global positive solution for any initial value $(x(0),y(0))\in {\mathbb {R}}^2_+$. This implies that model (2.3) has a unique local positive solution (*x*(*t*), *y*(*t*)) on $t\in [0,\tau _e)$, where $\tau _e$ is the explosion time. To show the solution is global, we only need to prove that $\tau _e=\infty$,* a.s.* To this end, let $n_0\ge 0$ be sufficiently large such that *x*(0), *y*(0) both lie within the interval $[\frac{1}{n_0},n_0]$. For each integer $n\ge 0$, define the stopping time as

$$\begin{aligned} \tau '_n=\inf \{t\in [0, \tau _e): \min \{x(t),y(t)\}\le \frac{1}{n} ~\text {or}~\max \{x(t),y(t)\}\ge n\}, \end{aligned}$$

where throughout this article, we set $\inf \emptyset =\infty$ (as usual $\emptyset$ denotes the empty set). Obviously, $\tau '_n$ is increasing as $n\rightarrow \infty$. Set $\tau '_{\infty }=\lim \limits _{n\rightarrow \infty }\tau '_n$, whence $\tau '_{\infty }\le \tau _e$, *a.s.* If we can prove $\tau '_{\infty }=\infty$, *a.s.*, then $\tau _{e}=\infty$ and $(x(t),y(t))\in {\mathbb {R}}^2_+$ for all $t\ge 0$, *a.s.* In other words, to complete the proof all we need to prove is that $\tau '_{\infty }=\infty$, *a.s.* We prove this by contradiction. If this assertion is false, then there exists a pair of constants $T>0$ and $\epsilon \in (0,1)$ such that

$$\begin{aligned} {\mathbb {P}}\{\tau '_{\infty }\le T\}>\epsilon . \end{aligned}$$

Therefore, there exists an integer $n_1\ge n_0$ such that for all $n\ge n_1$,

A.3 $$\begin{aligned} {\mathbb {P}}\{\tau '_n\le T\}\ge \epsilon . \end{aligned}$$

Define a $C^2$-function $V:{\mathbb {R}}^2_+\rightarrow {\mathbb {R}}_+$ by

$$\begin{aligned} V(x,y)=(x-\ell _1\ln x-\ell _1+\ell _1\ln \ell _1)+\frac{1}{e}(y-\ln y-1), \end{aligned}$$

where $\ell _1=\frac{ad}{ce}$. It is easy to verify that the function $V(x,y)\ge 0$ for all $(x,y)\in {\mathbb {R}}^2_+$. Applying Itô’s formula to model (2.3), we obtain that for $t\in [\tau _{2k}, \tau _{2k+1})$,

$$\begin{aligned} { LV}= & {} \left( 1-\frac{\ell _1}{x}\right) \left( bx(1-\frac{x}{K}) -\frac{cxy}{a+x}\right) +\frac{\ell _1\varepsilon _2^2}{2} \\&\quad +\frac{1}{e}\left( 1-\frac{1}{y}\right) \left( \frac{cey(p-y)}{a+x}-dy\right) +\frac{\varepsilon _2^2}{2e}\\= & {} \left( b+\frac{\ell _1b}{K}\right) x-\frac{b}{K}x^2 +\frac{1}{a+x}(-cy^2+(\ell _1c+cp+c)y-cp)\\&-\frac{cxy}{a+x}-\frac{d}{e}y-\ell _1\left( b-\frac{\varepsilon _2^2}{2}\right) +\frac{1}{e}\left( d+\frac{\varepsilon _2^2}{2}\right) \le M_1 \end{aligned}$$

and that for $t\in [\tau _{2k+1}, \tau _{2k+2})$,

$$\begin{aligned} { LV}= & {} \left( 1-\frac{\ell _1}{x}\right) \left( bx(1-\frac{x}{K})-\frac{cxy}{a+x}\right) +\frac{\ell _1\varepsilon _1^2}{2} +\frac{1}{e}\left( 1-\frac{1}{y}\right) \left( \frac{cexy}{a+x}-dy\right) +\frac{\varepsilon _1^2}{2e}\\= & {} \left( b+\frac{\ell _1b}{K}\right) x-\frac{b}{K}x^2+\frac{\ell _1cy}{a+x}-\frac{d}{e}y-\frac{cx}{a+x} -\ell _1\left( b-\frac{\varepsilon _1^2}{2}\right) +\frac{1}{e}\left( d+\frac{\varepsilon _1^2}{2}\right) \\\le & {} \left( b+\frac{\ell _1b}{K}\right) x-\frac{b}{K}x^2-\ell _1\left( b-\frac{\varepsilon _1^2}{2}\right) +\frac{1}{e}\left( d+\frac{\varepsilon _1^2}{2}\right) \le M_2. \end{aligned}$$

Therefore, we have that for $t\in [\tau _{2k}, \tau _{2k+1})$,

A.4 $$\begin{aligned} \mathrm {d}V(x,y) \le { LV}\mathrm {d}t+\varepsilon _2(x-\ell _1)\mathrm {d}B_1 +\frac{\varepsilon _2}{e}(y-1)\mathrm {d}B_2 \le M_1\mathrm {d}t+\varepsilon _2(x-\ell _1)\mathrm {d} B_1+\frac{\varepsilon _2}{e}(y-1)\mathrm {d}B_2 \end{aligned}$$

and that for $t\in [\tau _{2k+1}, \tau _{2k+2})$,

A.5 $$\begin{aligned} \mathrm {d}V(x,y) \le { LV}\mathrm {d}t+\varepsilon _1(x-\ell _1)\mathrm {d}B_1 +\frac{\varepsilon _1}{e}(y-1)\mathrm {d}B_2 \le M_2\mathrm {d}t+\varepsilon _1(x-\ell _1)\mathrm {d}B_1+\frac{\varepsilon _1}{e}(y-1) \mathrm {d}B_2. \end{aligned}$$

Without loss of generality, we assume that $(\tau '_n\wedge T)\in [\tau _{2m}, \tau _{2m+1})$ for some $k=m$ (same logic follows when $(\tau '_n\wedge T)\in [\tau _{2m+1}, \tau _{2m+2})$). This, together with (A.4) and (A.5), yields

$$\begin{aligned} \begin{aligned}&V(x(\tau '_n\wedge T),y(\tau '_n\wedge T))\\&\le V(x(0),y(0))+\sum _{k=0}^{m-1}\left( \int _{\tau _{2k}}^{\tau _{2k+1}} M_1dt+\int _{\tau _{2k}}^{\tau _{2k+1}}\varepsilon _2(x-\ell _1)dB_1+\int _{\tau _{2k}}^{\tau _{2k+1}}\frac{\varepsilon _2}{e}(y-1)dB_2 \right) \\&\quad +\sum _{k=0}^{m-1}\left( \int _{\tau _{2k+1}}^{\tau _{2k+2}} M_2dt+\int _{\tau _{2k+1}}^{\tau _{2k+2}}\varepsilon _1(x-\ell _1)dB_1+\int _{\tau _{2k+1}}^{\tau _{2k+2}}\frac{\varepsilon _1}{e}(y-1)dB_2 \right) \\&\quad +\int _{\tau _{2m}}^{\tau '_n\wedge T} M_1dt+\int _{\tau _{2m}}^{\tau '_n\wedge T}\varepsilon _2(x-\ell _1)dB_1+\int _{\tau _{2m}}^{\tau '_n\wedge T}\frac{\varepsilon _2}{e}(y-1)dB_2 . \end{aligned} \end{aligned}$$

Taking the expectations of the above inequality leads to

$$\begin{aligned} {\mathbb {E}}V(x(\tau '_n\wedge T),y(\tau '_n\wedge T))\le V(x(0),y(0))+M{\mathbb {E}}(\tau '_n\wedge T), \end{aligned}$$

where $M=\max \{M_1,M_2\}$. Noticing that ${\mathbb {E}}(\tau '_n\wedge T)\le T$, it then follows that

A.6 $$\begin{aligned} {\mathbb {E}}V(x(\tau '_n\wedge T),y(\tau '_n\wedge T))\le V(x(0),y(0))+MT. \end{aligned}$$

Let $\varOmega _n=\{\omega \in \varOmega :\tau '_n=\tau '_n(\omega )\le T\}$ for $n\ge n_1$ and in view of (A.3), we know that ${\mathbb {P}}(\varOmega _n)\ge \epsilon$. Notice that for every $\omega \in \varOmega _n$, we have either $x(\tau '_n, \omega )$ or $y(\tau '_n, \omega )$ equals either *n* or $\frac{1}{n}$. Hence $V(x(\tau '_n, \omega ), y(\tau '_n, \omega ))$ is no less than either

$$\begin{aligned}&(n-\ell _1\ln n-\ell _1+\ell _1\ln \ell _1)\wedge \frac{1}{e}(n-\ln n-1)~~\text {or}~~\left( \frac{1}{n}+\ell _1\ln n-\ell _1+\ell _1\ln \ell _1\right) \\&\quad \wedge \frac{1}{e}\left( \frac{1}{n}+\ln n-1\right) . \end{aligned}$$

It follows from (A.6) that

$$\begin{aligned} \begin{aligned}&V(x(0),y(0))+MT\ge {\mathbb {E}}[I_{\varOmega _{n}(\omega )}V(x(\tau '_n, \omega ), y(\tau '_n, \omega ))]\\&\quad \ge \epsilon \left[ (n-\ell _1-\ell _1\ln \frac{n}{\ell _1})\wedge \frac{1}{e}(n-\ln n-1)\right. \\&\left. \quad \wedge \left( \frac{1}{n}-\ell _1+\ell _1\ln (\ell _1n) \right) \wedge \frac{1}{e}\left( \frac{1}{n}+\ln n-1\right) \right] , \end{aligned} \end{aligned}$$

where $I_{\varOmega _n}$ denotes the indicator function of $\varOmega _n$. Letting $n\rightarrow \infty$, *a.s.*, then we obtain

$$\begin{aligned} \infty >V(x(0),y(0))+MT=\infty , \end{aligned}$$

which leads to the contradiction and thus we must have $\tau '_{\infty }=\infty$, *a.s.* This completes the proof. $\square$

### Remark A.1

In the proof of Theorem 2.1, we first argue that model (2.3) has a unique positive local solution, which suggests a strong solution that is pathwise unique, since this is the case for the two subproblems. However, the procedure used usually yields a weak solution with uniqueness in law, see sections 1.4 and 1.5 in Cherny and Engelbert (2005).

## B Stochastic Sensitivity and Confidence Domains

Consider a general nonlinear stochastic model

B.1 $$\begin{aligned} \dot{x}=f(x)+\varepsilon \sigma (x)\dot{w}, \end{aligned}$$

where *x* is an *n*-vector, *f*(*x*) is an *n*-vector function, $\sigma (x)$ is an $n\times n$-matrix-valued function, *w*(*t*) is an *n*-dimensional Brownian motion, and $\varepsilon$ is a scalar parameter of the noise intensity. We assume that the deterministic model corresponding to (B.1) $(\varepsilon =0)$ has a stable attractor.

Random trajectories of model (B.1) leave a deterministic attractor and form a corresponding stochastic attractor with stationary probability distribution $\rho (x,\varepsilon )$, which is a stationary solution of the corresponding Fokker-Planck equation. Technically, it is hard to acquire such a solution. For a weak noise, asymptotes based on the quasipotential $v(x)=\lim _{\varepsilon \rightarrow 0}\varepsilon ^{2}\log \rho (x,\varepsilon )$ are used (Freidlin and Wentzell 1984), and an approximation of $\rho (x,\varepsilon )$ can be written as

$$\begin{aligned} \rho (x,\varepsilon )\approx K\cdot \exp \bigg (-\frac{v(x)}{\varepsilon ^{2}}\bigg ). \end{aligned}$$

To approach *v*(*x*), we use the stochastic sensitivity functions (Bashkirtseva and Ryashko 2005).

We consider stochastically forced equilibria. We assume that the deterministic model (B.1) with $\varepsilon =0$ has a stable equilibrium ${\bar{x}}$. In this case, the following quadratic approximation of the quasipotential $v(x)\approx \frac{1}{2}(x-{\bar{x}}, V(x-{\bar{x}}))$ is used, one can obtain an asymptote of the stationary distribution in the Gaussian form:

$$\begin{aligned} \rho (x,\varepsilon )\approx K\cdot \exp \bigg (-\frac{(x-{\bar{x}}, V(x-{\bar{x}}))}{2\varepsilon ^{2}}\bigg ), \end{aligned}$$

where $\varepsilon ^{2}W=\varepsilon ^{2}V^{-1}$ is a covariance matrix. The stochastic sensitivity matrix *W* is a unique solution of the matrix equation

B.2 $$\begin{aligned} FW+WF^{T}=-S,F=\frac{\partial f}{\partial x}({\bar{x}}),S=GG^{T},G=\sigma ({\bar{x}}). \end{aligned}$$

This matrix characterizes a spatial arrangement and the size of the stationary distributed random states in the stochastic model (B.1) around the deterministic equilibrium ${\bar{x}}$. Using this matrix, one can construct confidence domains for the geometrical description of the stochastic attractors.

For two dimensional case, a confidence ellipse can be given by the following equation:

B.3 $$\begin{aligned} (x-{\bar{x}},W^{-1}(x-{\bar{x}}))=2k^{2}\varepsilon ^{2}, \end{aligned}$$

where $\varepsilon$ is a noise intensity, $k^{2}=-\ln (1-p)$, and *p* is a fiducial probability.

Now we consider that the deterministic model (B.1) with $\varepsilon =0$ has a stable limit cycle $\varGamma$ corresponding to *T*-periodic solution $x=\xi (t)$. Let $\varPi _{t}$ be a hyperplane which is orthogonal to the cycle $\varGamma$ at the point $\xi (t)(0\le t<T)$. In this case, for the Poincaré section $\varPi _{t}$ in the neighborhood of the point $\xi (t)$, the quadratic approximation of the quasipotential can be written as $v(x)\approx \frac{1}{2}(x-\xi (t), W^{+}(t)(x-\xi (t)))$. The corresponding Gaussian approximation of the stationary probabilistic distribution is as follows:

$$\begin{aligned} \rho (x,\varepsilon )\approx K\exp \bigg (-\frac{(x-\xi (t)^{T} W^{+}(t)(x-\xi (t))}{2\varepsilon ^{2}}\bigg ). \end{aligned}$$

Here, the stochastic sensitivity matrix *W*(*t*) of the cycle $\varGamma$ is a unique solution of the Lyapunov equation

$$\begin{aligned} \dot{W}=F(t)W +WF^{T}(t)+P(t)S(t)P(t) \end{aligned}$$

with conditions

$$\begin{aligned} W(0)=W(T),W(t)r(t)\equiv 0, \end{aligned}$$

where $F(t)=\frac{\partial f}{\partial x}(\xi (t))$, $S(t)=\sigma (\xi (t))\sigma ^{T}(\xi (t))$, $r(t)=f(\xi (t))$, and *P*(*t*) is a matrix of the orthogonal projection onto the hyperplane $\varPi _{t}$ (Ryashko 1996).

For two-dimensional case, the stochastic sensitivity matrix *W*(*t*) can be written as $W(t)=\mu (t)P(t)$. Here, $\mu (t)>0$ is a *T*-periodic scalar stochastic sensitivity function satisfying the following boundary problem:

B.4 $$\begin{aligned} {\dot{\mu }}=a(t)\mu +b(t),\mu (0)=\mu (T) \end{aligned}$$

with *T*-periodic coefficients

$$\begin{aligned} a(t)=\mu ^{T}(t)(F^{T}(t)+F(t))u(t), \quad b(t)=\mu ^{T}(t)S(t)u(t), \end{aligned}$$

where *u*(*t*) is a normalized vector orthogonal to $f(\xi (t))$.

The stochastic sensitivity function $\mu (t)$ allows us to construct a confidence band around the deterministic cycle $\varGamma$. For the line $\varPi _{t}$ that is orthogonal to the cycle at the point $\xi (t)$, a corresponding confidence interval is given by the following equation $(x-\xi (t))^{2} = 2k^{2}\varepsilon ^{2}\mu (t)$. Hence, the boundaries $x_{1,2}(t)$ of the confidence band can be written in an explicit parametrical form:

B.5 $$\begin{aligned} x_{1,2}(t)=\xi (t)\pm k\varepsilon \sqrt{2\mu (t)}u(t). \end{aligned}$$

Here, the parameter *k* is connected with the fiducial probability *p* by the formula $k = erf^{-1}(p)$, where $erf(x)=\frac{2}{\sqrt{\pi }}\int _{0}^{x}e^{-t^{2}}dt$ is the error function.

The stochastic sensitivity function technique was successfully applied to analyze noise-induced transitions in a stoichiometric producer–grazer model and to construct confidence domains.
